# Engagement With a Mobile Chat-Based Intervention for Smoking Cessation: A Secondary Analysis of a Randomized Clinical Trial

**DOI:** 10.1001/jamanetworkopen.2024.17796

**Published:** 2024-06-26

**Authors:** Yajie Li, Tzu Tsun Luk, Yee Tak Derek Cheung, Shengzhi Zhao, Yingpei Zeng, Henry Sau Chai Tong, Vienna Wai Yin Lai, Man Ping Wang

**Affiliations:** 1 School of Nursing, The University of Hong Kong, Hong Kong Special Administrative Region, China; 2 Hong Kong Council on Smoking and Health, Hong Kong Special Administrative Region, China

## Abstract

**Importance:**

Determining how individuals engage with digital health interventions over time is crucial to understand and optimize intervention outcomes.

**Objective:**

To identify the engagement trajectories with a mobile chat-based smoking cessation intervention and examine its association with biochemically validated abstinence.

**Design, Setting, and Participants:**

A secondary analysis of a pragmatic, cluster randomized clinical trial conducted in Hong Kong with 6-month follow-up. From June 18 to September 30, 2017, 624 adult daily smokers were recruited from 34 community sites randomized to the intervention group. Data were analyzed from March 6 to October 30, 2023.

**Intervention:**

Chat-based cessation support delivered by a live counselor via a mobile instant messaging app for 3 months from baseline.

**Main Outcomes and Measures:**

Group-based trajectory modeling was used to identify engagement trajectories using the participants’ weekly responses to the messages from the counselor over the 3-month intervention period. The outcome measures were biochemically validated tobacco abstinence at 3-month (end of treatment) and 6-month follow-ups. Covariates included sex, age, educational level, nicotine dependence, past quit attempt, and intention to quit at baseline.

**Results:**

Of 624 participants included in the analysis, 479 were male (76.8%), and the mean (SD) age was 42.1 (16.2) years. Four distinct engagement trajectories were identified: low engagement group (447 [71.6%]), where participants maintained very low engagement throughout; rapid-declining group (86 [13.8%]), where participants began with moderate engagement and rapidly decreased to a low level; gradual-declining group (58 [9.3%]), where participants had high initial engagement and gradually decreased to a moderate level; and high engagement group (58 [5.3%]), where participants maintained high engagement throughout. Compared with the low engagement group, the 6-month validated abstinence rates were significantly higher in the rapid-declining group (adjusted relative risk [ARR], 3.30; 95% CI, 1.39-7.81), gradual-declining group (ARR, 5.17; 95% CI, 2.21-12.11), and high engagement group (ARR, 4.98; 95% CI, 1.82-13.60). The corresponding ARRs (95% CI) of 3-month validated abstinence were 4.03 (95% CI, 1.53-10.59), 5.25 (95% CI, 1.98-13.88), and 9.23 (95% CI, 3.29-25.86).

**Conclusions and Relevance:**

The findings of this study suggest that higher levels of engagement with the chat-based smoking cessation intervention were associated with greater biochemically validated tobacco abstinence. Improving engagement with digital interventions may increase intervention benefits.

**Trial Registration:**

ClinicalTrials.gov Identifier: NCT03182790

## Key Points

**Question:**

How does engagement with a mobile chat-based intervention affect smoking abstinence among cigarette smokers?

**Findings:**

In this secondary analysis of a cluster randomized clinical trial involving 624 cigarette smokers receiving chat-based smoking cessation support via mobile instant messaging for 3 months, 4 distinct engagement trajectories were identified. Higher level of engagement was associated with greater biochemically validated smoking abstinence at 3- and 6-month follow-ups.

**Meaning:**

The findings of this study suggest the importance of assessing engagement patterns for a comprehensive understanding of intervention and improving user engagement.

## Teaser

This secondary analysis of a randomized clinical trial examines the use of support delivered by a live counselor via a mobile instant messaging for smoking cessation among individuals in Hong Kong.

## Introduction

The effectiveness of a digital health intervention is contingent on the users’ level of participation, involvement, and interaction, which vary among individuals and evolve over time.^1,2,3^ Intervention engagement is commonly conceptualized by the use (eg, amount, frequency, depth, and duration) and subjective experience (eg, user’s interest, attention, and affect) with the intervention.^2,4,5^ Systematic reviews reported that intervention engagement is associated with positive health behavioral changes and mental health improvements.^6,7^ However, as described by the law of attrition, a substantial proportion of users of digital health interventions drop out or stop using the interventions prematurely,^8^ which complicates the interpretation of intervention efficacy.^2,8^

Mobile phone–based interventions, particularly text messaging, have been proven useful in promoting smoking cessation.^9^ Some text messaging–based interventions incorporated interactive features, allowing users to respond to messages (eg, by typing key words) to elicit on-demand support. Trajectory modeling, a statistical method for identifying patterns of change over time,^10,11^ has been applied to understand the level of engagement with text messaging–based cessation programs, including SmokefreeVET^12^ and SmokefreeTXT^13^ in the US. By analyzing weekly text responses from users, 5 distinct trajectories were identified in SmokefreeVET and 4 in SmokefreeTXT, with greater short-term (<6 weeks) self-reported quitting observed in users with higher engagement.^12,13^ Nevertheless, to our knowledge, no study has reported longer-term abstinence outcomes (≥6 months) with biochemical validation, which is more rigorous for evaluating intervention outcomes.^14^

Given the widespread ownership of smartphones, we developed a novel chat-based intervention that allows a counselor to interact with a smoker in real-time and provide personalized cessation support via mobile instant messaging apps.^15^ A subsequent cluster randomized clinical trial found the chat-based intervention more effective than brief intervention alone in promoting biochemically validated abstinence at 3 months (odds ratio [OR], 1.95) and 6 months (OR, 1.68) in community-based smokers.^16^ However, how participants engage in the chat-based intervention and its influence on abstinence outcomes have remained unclear. This study aimed to examine the trajectories of engagement in the chat-based intervention and its associations with long-term biochemically validated abstinence.

## Methods

### Study Design

This study was a post hoc secondary analysis of the 2-arm, parallel, pragmatic, cluster randomized clinical trial (protocol available in Supplement 1) nested in the Quit to Win Smoke-Free Community Campaign in 2017, a smoking cessation contest organized by the Hong Kong Council on Smoking and Health. Details of the trial design^17^ and main results^16^ have been reported. In this trial, 68 community sites were randomized (1:1) to the intervention group (n = 34), in which participants received chat-based instant messaging support for 3 months combined with brief cessation intervention, or to the control group (n = 34), in which participants received brief cessation advice only. Participants did not receive financial compensation. The primary outcome was biochemically validated tobacco abstinence at 6 months after baseline. The study followed the Consolidated Standards of Reporting Trials (CONSORT) reporting guideline. Ethical approval was obtained from the institutional review board of the University of Hong Kong and the Hospital Authority Hong Kong West Cluster.

### Participants

From June 18 to September 30, 2017, participants were proactively recruited from 68 community sites (eg, vicinities of shopping malls, housing estates, and transport facilities) across all 18 districts in Hong Kong. Eligible participants were daily cigarette smokers in the preceding 3 months (verified by an exhaled carbon monoxide level of ≥4 ppm), aged 18 years or older, intended to quit or reduce smoking, owned a smartphone with an instant messaging app installed, had no physical or cognitive communication barriers, and were not participating in other smoking cessation programs. The trial participants were largely comparable to the daily cigarette smokers in the general population in terms of sex, age, and daily cigarette consumption.^16^ The present study analyzed data from 624 participants recruited from 34 community sites randomized to the intervention group.

### Intervention

Participants in the intervention group received brief cessation advice at baseline and chat-based cessation support via mobile instant messaging app for 3 months from baseline. Details of the chat-based intervention were described elsewhere.^15,16,17^ Briefly, participants received a total of 19 instant messages from the counselors during the 3-month intervention period, with a frequency of at least 1 message per week. These messages included generic information about the benefits of smoking cessation, methods to manage craving, smoking cessation services, and reminders to participate in the telephone follow-ups. By responding to the messages, the participants could initiate a chat-based support session. During these sessions, the counselor interacted with participants and provided cessation advice guided by Acceptance and Commitment Therapy.^18^ Based on the progress and needs of the participants, the counselor also applied behavior change techniques to enhance motivation to quit, improve self-regulatory capacities, use adjuvant cessation activities, and facilitate interaction with participants.^19^ Participants were also offered referral to a local cessation service to receive more-intensive cessation support (eg, pharmacotherapy). Due to resource constraints, all chat conversations occurred during office hours on working days. Participants were informed about the availability of the counselor at the beginning of the intervention.

### Measures

Baseline data were collected in-person using questionnaires, whereas follow-up data were collected via telephone surveys. Baseline measures included demographic characteristics (ie, sex, age, and educational level), cigarette consumption, nicotine dependence assessed by the Heaviness of Smoking Index (scores ranged from 0 to 6, with higher scores indicating greater nicotine dependence), past quit attempts, intention to quit, and perceptions (importance, difficulty, and confidence) of quitting (scores ranged from 0 to 10, with higher scores indicating greater perceived importance, difficulty, or confidence).

The main outcome was biochemically validated tobacco abstinence at 6 months after randomization, confirmed by an exhaled carbon monoxide level of less than 4 ppm (Bedfont piCO Smokerlyzer; Bedfont Scientific)^20^ and salivary cotinine level of less than 10 ng/mL (to convert to nanomoles per liter, multiply by 5.675) (NicAlert; Nymox Pharmaceutical).^21^ The main secondary outcome was biochemically validated tobacco abstinence at 3 months (end of intervention). All participants who reported having abstained from tobacco use for at least 7 days during the 3- or 6-month follow-ups were invited to participate in the validation, with a small cash incentive of approximately US $64 (HK$500) to promote participation. Other follow-up measures included self-reported 7-day point prevalence tobacco abstinence and use of any smoking cessation services from baseline to 3- and 6-month follow-ups.

To assess intervention engagement, we retrieved all mobile instant messaging app chat dialogues and recorded the number of participants’ responses to each of the 19 push messages from the counselors. Responses included text, emojis, voice messages, and the combination of these. Using a similar approach applied in previous studies on text messaging programs,^12,13^ weekly responses were used to delineate the engagement trajectories. For each week during the 3-month intervention period, a binary variable was created based on whether participants responded to the messages of that week, representing the participants’ engagement with the chat-based support session.

### Statistical Analysis

Data were analyzed from March 6 to October 30, 2023. Stata, version 16 (StataCorp LLC) was used for statistical analysis. Group-based trajectory modeling, a type of latent class modeling, was adopted to identify the engagement trajectories with the chat-based intervention. We used the plugin in Stata, traj, to group participants with a similar pattern of weekly response to the intervention over time.^22^ The hypotheses regarding trajectory shapes and the number of trajectory groups were tested with maximum likelihood estimation. All possible models with a polynomial order of 0 to 4 and the number of trajectory groups of 1 to 5 were built. We selected the model with the highest (least negative) bayesian information criterion (BIC) and sufficient sample size (at least 5% of the total) in each trajectory group.^10^ Model adequacy is indicated by (1) an average group posterior probability of greater than 0.7 for each group, (2) an odds of correct classification of 5 or more for all groups, and (3) a close correspondence between the estimated group probabilities and the proportion of group assignment using the maximum posterior probability assignment rule.^23^

One-way analysis of variance and χ^2^ tests were used to compare the baseline characteristics across participants in different engagement trajectories identified by group-based trajectory modeling. Ordinal logistic regression was used to examine the associations between baseline characteristics and trajectory group membership, which is treated as an ordinal variable with increasing engagement level. An insignificant Brant test (*P* > .99) supported the proportional odds assumption.

Poisson regression with robust variance was performed to estimate the relative risk (RR) of abstinence outcomes by engagement trajectories, adjusting for sex, age, nicotine dependence, intention to quit, and past quit attempts at baseline.^24^ As a sensitivity analysis, we additionally adjusted for the perceptions of quitting and any use of cessation service during the intervention period. Participants who did not complete follow-up were assumed to be nonabstinent and did not use any cessation service. Complete case analyses were also conducted by excluding participants without follow-up data. All statistical tests were 2-sided, with *P* < .05 indicating statistical significance.

## Results

### Participant Characteristics

Of 1254 participants in the trial, 624 were randomized to the intervention group and included in this secondary analysis (eFigure in Supplement 2). The baseline characteristics of all randomized participants are presented in eTable 1 in Supplement 2. Among the 624 participants, 479 were male (76.8%) and 145 were female (23.2%), with a mean (SD) age of 42.1 (16.2) years (Table 1).

**Table 1.** Baseline Characteristics of Participants by Engagement Trajectories

Characteristic	Total, No. (%)^a^	Engagement trajectories, No. (%)				*P* value^a^
		Low (n = 447)	Rapid declining (n = 86)	Gradual declining (n = 58)	High (n = 33)	
Sex						
Male	479 (76.8)	347 (77.6)	60 (67.8)	46 (79.3)	26 (78.8)	.42
Female	145 (23.2)	100 (22.4)	26 (30.2)	12 (20.7)	7 (21.2)	
Age, mean (SD), y	42.1 (16.2)	41.6 (17.3)	42.1 (12.7)	42.4 (12.4)	47.5 (15.7)	.30
Age group, y						
18-29	125 (22.6)	100 (25.9)	14 (16.9)	8 (14.6)	3 (10.0)	.15
30-39	140 (25.3)	97 (25.1)	21 (25.3)	16 (29.1)	6 (20.0)	
40-49	125 (22.6)	77 (20.0)	21 (25.3)	17 (30.9)	10 (33.3)	
≥50	164 (29.6)	112 (29.0)	27 (32.5)	14 (25.5)	11 (36.7)	
Educational level						
Primary or below	26 (6.6)	18 (7.2)	3 (4.4)	4 (8.5)	1 (3.3)	.80
Secondary	284 (72.1)	181 (72.7)	50 (73.5)	30 (63.8)	23(76.7)	
Tertiary	84 (21.3)	50 (20.1)	15 (22.1)	13 (27.7)	6 (20.0)	
Daily cigarette consumptions, mean (SD)	13.8 (8.3)	13.8 (8.7)	14.3 (7.5)	12.5 (7.1)	14.2 (7.1)	.62
Nicotine dependence^b^						
Low	295 (50.8)	210 (51.0)	35 (43.2)	31 (54.4)	19 (61.3)	.32
Moderate/high	286 (49.2)	202 (49.0)	46 (56.8)	26 (45.6)	21 (38.7)	
Intention to quit in 30 d						
Yes	223 (37.2)	123 (28.7)	40 (48.8)	39 (68.4)	21 (63.6)	<.001
No	377 (62.8)	305 (71.3)	42 (51.2)	18 (31.6)	12 (36.4)	
Past quit attempt						
Never	273 (47.1)	205 (50.0)	39 (47.6)	19 (33.9)	10 (32.3)	.09
Beyond past 12 mo	237 (40.9)	164 (39.9)	32 (39.0)	27 (48.2)	14 (45.2)	
Within past 12 mo	70 (12.1)	42 (10.2)	11 (13.4)	10 (7.9)	7 (22.6)	
Perceptions of quitting, mean (SD)^c^						
Importance	7.2 (2.1)	6.9 (2.1)	8.0 (2.0)	8.2 (2.0)	8.2 (1.5)	<.001
Difficulty	7.1 (2.2)	7.0 (2.1)	7.3 (2.3)	7.5 (2.4)	7.4 (2.5)	.23
Confidence	6.0 (2.2)	5.8 (2.1)	6.2 (2.2)	6.5 (2.1)	7.1 (2.4)	.001

^a^ *P* values were calculated by χ^2^ test or 1-way analysis of variance as appropriate.

^b^ Measured by the Heaviness of Smoking Index; scores range from 0 to 6, with higher scores indicating greater nicotine dependence.

^c^ Scores ranged from 0 to 10, with higher scores indicating higher perceived importance, difficulty, or confidence.

### Model Selection

eTable 2 in Supplement 2 reports the 5 best models of all possible combinations of polynomial order and number of groups, with the 4-group model (polynomial order, 2111) yielding the greatest BIC for the total number of observations (BIC, −1806.98) and the 5-group model (polynomial order, 11121) yielding the greatest BIC for the total number of participants (BIC, −1790.74). However, only the 4-group model achieved the minimum group size of at least 5% for each group and was thus selected for further analysis. The model adequacy was supported by an average group posterior probability of 0.86 or above for each group, odds of correct classification of 7.35 or above, and close correspondence between the estimated group probabilities and the proportion of group assignment (eTable 3 in Supplement 2).

### Engagement Trajectories

The Figure shows the 4 distinct engagement trajectories identified with increasing levels of engagement from bottom to top. The trajectories were labeled as low engagement (estimated group probability of 68.6%), representing participants who maintained low to no engagement throughout the 3-month intervention period; rapid-declining engagement (16.5%), representing participants who began with moderate engagement that rapidly declined and maintained at a low level; gradual-declining engagement (9.2%), representing participants with a high initial engagement that gradually declined to a moderate level; and high engagement (5.6%), representing participants who maintained high engagement throughout.

**Figure.**
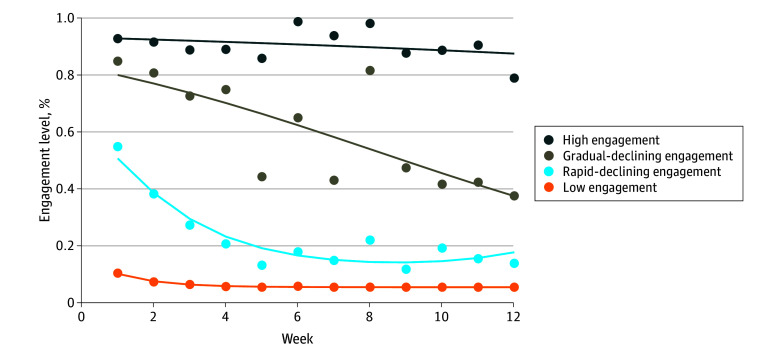
Engagement Trajectories Identified by Group-Based Trajectory Modeling

### Baseline Characteristics

Table 1 reports the actual proportion assigned to each group with the maximum probability rule: 71.6% (n = 447) was assigned to the low-engagement group, followed by 13.8% (n = 86) to the rapid-declining engagement group, 9.3% (n = 58) to the gradual-declining engagement group, and 5.3% (n = 33) to the high-engagement group. The proportion of participants with an intention to quit in 30 days generally increased from the low-engagement group (28.7%) to the high-engagement group (63.6%) (*P* < .001). Similarly, mean (SD) perceived importance (8.2 [1.5] vs 6.9 [2.1]; *P* < .001) and confidence (7.1 [2.4] vs 5.8 [2.1]; *P* = .001) were significantly higher in the high-engagement group compared with the low-engagement group.

### Factors Associated With Higher Engagement Level

Table 2 reports that older age, intention to quit in 30 days, quit attempt within the past 12 months, and perceived importance, difficulty, and confidence of quitting were associated with higher levels of engagement. The findings were significant after adjusting for other baseline characteristics, except for the perceptions of quitting.

**Table 2.** Associations of Baseline Characteristics With the Level of Engagement

Characteristic	OR (95% CI)	*P* value	Adjusted OR (95% CI)^a^	*P* value
Sex				
Male	1 [Reference]	NA	1 [Reference]	NA
Female	1.12 (0.75-1.66)	.58	1.49 (0.86-2.60)	.16
Age group, y				
18-29	1 [Reference]	NA	1 [Reference]	NA
30-39	1.78 (1.02-3.12)	.04	1.74 (0.83-3.65)	.14
40-49	2.57 (1.47-4.49)	.001	3.04 (1.42-6.50)	.004
≥50	1.87 (1.09-3.22)	.02	3.63 (1.63-8.10)	.002
Educational level				
Primary or below	1 [Reference]	NA	1 [Reference]	NA
Secondary	1.25 (0.53-2.92)	.61	0.89 (0.33-2.35)	.81
Tertiary	1.48 (0.59-3.71)	.40	1.33 (0.45-3.96)	.61
Daily cigarette consumptions	1.00 (0.98-1.02)	.80	0.99 (0.96-1.03)	.69
Nicotine dependence^b^				
Low	1 [Reference]	NA	1 [Reference]	NA
Moderate/high	0.97 (0.68-1.38)	.85	0.62 (0.35-1.12)	.11
Intention to quit in 30 d				
No	1 [Reference]	NA	1 [Reference]	NA
Yes	3.55 (2.47-5.09)	<.001	2.60 (1.55-4.35)	<.001
Past quit attempt				
Never	1 [Reference]	NA	1 [Reference]	NA
Beyond past 12 mo	1.40 (0.95-2.05)	.09	1.46 (0.87-2.44)	.15
Within past 12 mo	2.15 (1.25-3.67)	.005	2.05 (1.01-4.18)	.054
Perceptions of quitting^c^				
Importance	1.34 (1.22-1.48)	<.001	1.13 (0.99-1.29)	.08
Difficulty	1.09 (1.00-1.19)	.04	1.06 (0.95-1.18)	.29
Confidence	1.17 (1.08-1.27)	<.001	1.10 (0.97-1.23)	.13

Abbreviations: NA, not applicable; OR, odds ratio.

^a^ Adjusted for other variables in the table.

^b^ Measured by the Heaviness of Smoking Index; scores range from 0 to 6, with higher scores indicating greater nicotine dependence.

^c^ Scores range from 0 to 10, with higher scores indicating higher perceived importance, difficulty, or confidence.

### Outcomes by Engagement Trajectories

The overall retention rate was 69.4% (n = 433) at 3 months and 80.1% (n = 500) at 6 months, which were significantly lower in the low-engagement group than the other 3 groups (eTable 4 in Supplement 2). Table 3 reports that 6-month validated abstinence rates tend to be in groups with greater engagement: 24.2% in the high-engagement group, 27.6% in the gradual-declining group, 15.1% in the rapid-declining group, and 3.6% in the low-engagement group. The corresponding abstinence rates at 3 months were 33.3% in the high-engagement group, 22.4% in the gradual-declining group, 15.1% in the rapid-declining group, and 2.9% in the low-engagement group. Compared with the low-engagement group, the 6-month validated smoking abstinence rate was significantly higher with the rapid-declining engagement group (adjusted RR [ARR], 3.30; 95% CI, 1.39-7.81; *P* = .007), gradual-declining engagement group (ARR, 5.17; 95% CI, 2.21-12.11; *P* < .001), and high-engagement group (ARR, 4.98; 95% CI, 1.82-13.60; *P* = .002). The corresponding ARRs of 3 months were 4.03 (95% CI, 1.53-10.59), 5.25 (95% CI, 1.98-13.88), and 9.23 (95% CI, 3.29-25.86). The associations between self-reported 7-day point prevalence tobacco abstinence and engagement trajectories were consistent with those observed in validated abstinence. eTable 5 in Supplement 2 reports the associations attenuated, but the findings were significant after additionally adjusting for perceptions of quitting and use of cessation service. eTable 6 in Supplement 2 reports that complete case analyses yielded similar results.

**Table 3.** Associations of Engagement Trajectories With Smoking Abstinence^a^

Variable	6-mo Follow-up			3-mo Follow-up		
	No./total No. (%)	RR (95% CI)	Adjusted RR (95% CI)^b^	No./total No. (%)	RR (95% CI)	Adjusted RR (95% CI)^b^
**Biochemically validated abstinence**						
Low engagement	16/447 (3.6)	1 [Reference]	1 [Reference]	13/447 (2.9)	1 [Reference]	1 [Reference]
Rapid declining	13/86 (15.1)	4.22 (2.03-8.78)	3.30 (1.39-7.81)	13/86 (15.1)	5.20 (2.41-11.21)	4.03 (1.53-10.59)
Gradual declining	16/58 (27.6)	7.71 (3.85-15.41)	5.17 (2.21-12.11)	13/58 (22.4)	7.71 (3.57-16.62)	5.25 (1.98-13.88)
High engagement	8/33 (24.2)	6.77 (2.90-15.83)	4.98 (1.82-13.60)	11/33 (33.3)	11.46 (5.13-25.58)	9.23 (3.29-25.86)
**Self-reported 7-d point prevalence abstinence**						
Low engagement	36/447 (8.1)	1 [Reference]	1 [Reference]	45/447 (10.1)	1 [Reference]	1 [Reference]
Rapid declining	21/86 (24.4)	1.84 (1.14-2.96)	1.61 (0.88-2.95)	23/86 (26.7)	2.66 (1.61-4.39)	2.36 (1.21-4.60)
Gradual declining	16/58 (27.6)	2.61 (1.61-4.23)	2.25 (1.22-4.13)	18/58 (31.0)	3.08 (1.78-5.33)	2.55 (1.26-5.18)
High engagement	12/33 (36.4)	2.71 (1.49-4.91)	2.12 (1.03-4.38)	12/33 (36.4)	3.61 (1.91-6.83)	2.68 (1.20-6.00)

Abbreviation: RR, relative risk.

^a^ Participants with missing data were imputed as smoking.

^b^ Sex, age, educational level, nicotine dependence, intention to quit in 30 days, and past quit attempt at baseline were adjusted.

## Discussion

To our knowledge, this is the first study to examine the engagement trajectories in a mobile chat-based intervention for smoking cessation and their associations with smoking abstinence. Using group-based trajectory modeling, we identified 4 distinct trajectories and observed a smaller number of participants with higher levels of engagement. Older age, intention to quit in 30 days, and past quit attempt, but not nicotine dependence, were associated with higher engagement level, adjusting for other baseline characteristics. Higher engagement level was associated with higher biochemically validated abstinence at 3 months (end of intervention) and 6 months. Associations were also noted after adjusting for important predictors of quitting, including nicotine dependence, past quit attempt, and intention to quit.^24^

Direct comparison of our study with prior studies on engagement trajectories in digital health cessation interventions was challenging due to differences in the delivery platform, intervention duration, sample characteristics, and settings. Previous studies of text messaging,^12,13^ web-based interventions,^25^ and smartphone apps^26^ for smoking cessation have reported varying numbers of trajectory groups, ranging from 2 to 5, with different shapes of trajectories. Nevertheless, there were some common findings. First, several studies showed that most participants belonged to the group with the lowest engagement trajectory (range, 49.3%-65.3%), while those who were most engaged constituted the lowest proportion (range, 12.2%-34.7%).^12,13,25,26^ Our findings agree with these studies by showing that 71.6% of the participants belonged to the low-engagement group vs 5.6% in the high-engagement group. These findings support the notion that most users of digital health interventions do not use the intervention.^8^

Second, our findings corroborate previous research that showed an association between engagement and abstinence. Users who were highly engaged (ie, more weekly texts sent by users) in SmokefreeVET or SmokefreeTXT were significantly more likely to report short-term (<6 weeks) abstinence.^12,13^ Similarly, studies of web-based programs (WebQuit and Smokefree.gov) and a cessation app (iCanQuit) found higher self-reported 30-day point prevalence abstinence (OR, 1.48-4.97) in participants with higher engagement (ie, more web page and application openings).^25,26,27^ Our study strengthens the literature by using 6-month biochemically validated abstinence as the outcome measure, which is considered the standard in smoking cessation studies.^14^ The results from self-reported quitting were also consistent. Participants with a modest level of engagement (ie, rapidly-declining group) already showed a much higher abstinence rate compared with the low-engagement group (15.1% vs 3.6% for 6-month validated abstinence). This suggests implementing strategies to encourage the vast majority of participants with low engagement to use the program could greatly enhance the intervention benefits. Given our findings that intention to quit within 30 days was the only modifiable factor associated with greater engagement, integrating gamification components^28^ and financial incentives^29^ into the intervention model could be considered for promoting smokers’ motivation and intervention engagement.

While the high-engagement group exhibited the largest validated quit rate at 3 months (33.3%), the quit rate was reduced to 24.2% at 6 months, which was lower than that in the gradual-declining group (27.6%). A potential explanation was that participants who maintained high engagement throughout the intervention period tended to be more reliant on the support than those who gradually disengaged. The withdrawal of support after the end of the intervention could potentially compromise an individual’s ability to sustain quitting efforts. While speculative, these findings suggest the need for extended support to maximize long-term quitting success. Further research is warranted to evaluate the different duration of the chat-based intervention in quitting outcomes.

### Limitations

This study had some limitations. First, the identification of engagement trajectories was based on participants’ weekly responses to the counselors’ messages during the 3-month intervention period. This quantitative approach did not consider the subjective experiences or actual content of the participants’ responses. Further studies are warranted to analyze the conversation between the participant and counselor, including the behavioral change techniques involved, to gain a more comprehensive understanding of engagement. Second, albeit less likely, it is possible that abstinence outcomes can influence intervention engagement. Future research using a cross-lagged method with repeated measurement of abstinence and engagement can better understand the potential bidirectional nature of their association. Third, participants lost to follow-up were assumed to be nonabstinent, which is a standard approach used in smoking cessation studies.^30^ While this gives a more conservative estimate of the abstinence rate, complete case analyses yielded similar results. Fourth, although we adjusted for well-established predictors of smoking cessation, including nicotine dependence, intention to quit, and past quit attempt,^24^ residual and unmeasured confounding could not be excluded. Fifth, our sample included mostly men (76.8%), which reflects the male predominance of smoking in Hong Kong and other Asian countries. While the generalizability of the findings to populations in which women smoke is more common is uncertain, prior text messaging-based programs have not shown clear differences in engagement between sexes.^12,13^

## Conclusions

By applying group-based trajectory modeling, this secondary analysis of a randomized clinical trial identified 4 engagement trajectories of a mobile chat-based intervention for smoking cessation. A higher level of engagement was associated with greater biochemically validated tobacco abstinence. The findings suggest that the intervention involving strengthening users’ motivation to quit and extending the treatment duration may improve the abstinence outcomes.

## Notes

Supplement 1.
Protocol

Supplement 2. **eFigure.** Participant Flow Diagram **eTable 1.** Baseline Characteristics of All Randomized Participants **eTable 2.** Model Selection Results **eTable 3.** Model Diagnostics **eTable 4.** Retention Rate by Engagement Trajectories **eTable 5.** Associations of Engagement Trajectories With Smoking Abstinence **eTable 6.** Associations of Engagement Trajectories With Smoking Abstinence by Complete Case Analyses

Supplement 3.
Data Sharing Statement
