# Supplementary material for: Engagement With a Mobile Chat-Based Intervention for Smoking Cessation: A Secondary Analysis of a Randomized Clinical Trial
Source: JAMA Netw Open. 2024 Jun 26;7(6):e2417796. doi: 10.1001/jamanetworkopen.2024.17796 (PMC11208971; doi:10.1001/jamanetworkopen.2024.17796)
Supplement: Supplement 1. — Protocol [file jamanetwopen-e2417796-s001.pdf]

STUDY PROTOCOL (Version 3)

1. Project Title:

Building capacity and promoting smoking cessation in the community via "Quit to Win" Contest 2017: a single-blind cluster randomized controlled trial on a combined "cocktail" intervention of brief advice, instant messaging and active referral (AIR) to increase abstinence

2. Investigators

Principal Investigator

Dr. MP Wang, Assistant Professor, School of Nursing, HKU

Co-investigators

Prof. TH Lam, Chair Professor, School of Public Health, HKU
Dr. Derek YT Cheung, Research Assistant Professor, School of Nursing, HKU
Dr. William HC Li, Associate Professor, School of Nursing, HKU

3. Study sites

The study will be conducted in the community of all 18 District Council districts in Hong Kong.

4. Aims of the study

The aim of this project is to promote and evaluate an innovative community-based smoking cessation "cocktail" intervention through the "Quit to Win" Contest organized in all the 18 districts of Hong Kong. The specific objectives of the study are:

(1) To build capacity in the community on smoking cessation through a train the trainer (TTT) programme;
(2) To empower the community organizations at the district level to raise the awareness of the harms of smoking and the importance of smoking cessation and reach smokers in the community;
(3) To test, by a 2-arm RCT, the effectiveness of a combined intervention of face-to-face brief cessation advice (AWARD), semi-personalised interactive instant SC messaging plus active referral to smoking cessation (SC) services (AIR) compared with control subjects among current smokers who joined the contest;

(4) To evaluate the process and outcome of the recruitment of smokers through the recruitment activities by telephone surveys and qualitative interview

(5) To conduct qualitative interviews with quitters and non-quitters participated in the RCT to explore their experience on the interventions.

5. Outcome measure(s):

a) Building up the capacity of community-based smoking cessation intervention

The outcomes are to increase the knowledge, attitudes and competence of community workers in providing brief smoking cessation intervention.

b) To evaluate the process and outcome of the recruitment of smokers in the community

Recruitment input: (i) number of staff/helpers from non-government organizations (NGOs) and The University of Hong Kong (HKU) trained to participate in recruitment and providing community-based smoking cessation services; (ii) number of leaflets and self-help materials distributed in the recruitment activities.

Recruitment outcomes: (i) number of recruitment activities organized under the Quit to Win Contest; (ii) number of people, including smokers and non-smokers, reached in all the recruitment sessions; (iv) number of eligible participants enrolled into the Contest;

c) Testing the effectiveness of two innovative smoking cessation interventions

The primary outcome is biochemically validated abstinence at 6-month after baseline. Secondary outcomes include biochemically validated smoking cessation at 3-month, self-reported past 7-day point prevalence abstinence, smoking cessation services use; biochemically validated smoking cessation; and smoking reduction (50% or above reduction in cigarette consumption compared with baseline) at 3- and 6-month. Smoking cessation services use included several indicators: calling a hotline, booking an appointment, smoking cessation clinic attendance, counselling session attendance, and other indicators to be further specified after liaison with the existing services (e.g. services providers' records on services utilization).

6. Estimated duration and commencement date

Proposed starting date: 1 June 2017

Proposed study completion date: 30 May 2018
Expected final report date: 30 September 2018

7. Scientific/historical background

Smoking and second-hand smoke in Hong Kong

Although smoking prevalence is decreasing in Hong Kong, there are still 641,300 daily smokers (10.5%) (Census and Statistics Department, 2016) and half will be killed by smoking (Lam, 2012) which accounts for over 7,000 deaths per year (Lam, Ho, Hedley, Mak, & Peto, 2001). Smoking also accounts for a large amount of medical cost, long-term care and productivity loss of US$688 million (0.6% Hong Kong GDP) (Census & Statistics Department, 2001; McGhee et al., 2006). Smoking is a highly addictive behavior and it is difficult for smokers with strong nicotine dependence to quit without assistance. On the other hand, reaching and helping the many smokers who have no intention to quit is a challenge, because they are unlikely to seek professional help from smoking cessation services.

Previous Quit to Win Contest findings

The Quit and Win programme provides an opportunity to reach and encourage a large group of smokers to make quit attempt and maintain abstinence. The Quit and Win model posits that smokers participating in the contest will have higher motivation to quit with incentives and better social support (Cahill & Perera, 2011). Studies have found that such quitting contests or incentive programs appeared to reach a large number of smokers and demonstrated a significantly higher quit rate for the quit and win group than for the control group (Cahill & Perera, 2008).

In 2009, we conducted a 3-arm RCT to compare the effectiveness of a 3-minute brief telephone advice, 8 mobile phone (SMS) messages and usual care of smoking cessation self-help booklet (Chan, 2011). More than one thousand participants were successfully recruited in 1.5 months with an overall self-reported quit rate of 21.6% at 6 months. However, the 2 interventions groups did not show a higher quit rate than the control group. In the Quit to Win Contest 2010, we compared the effectiveness of an on-site face-to-face brief smoking cessation advice (intervention) with self-help booklet (control) on quit rate and changes in smoking behavior. Once again, we recruited over one thousand participants in 2.5 months. A marginally significant ($p = 0.08$) higher quit rate was observed in the intervention group (18.4%) compared with

the control group (13.8%) at the 6-month follow-up (Wong & Chan, 2012). The Quit to Win Contest 2012 studied on the effectiveness of the on-site counselling with telephone boosters and health education card was theoretically based on the Health Action Process Approach (HAPA) for the intervention group (Schwarzer, 2008) and the SMS intervention group who received 16 SMS about cessation advice and motivation were compared with the control group. The HAPA suggests that one's intention of behavior change can be fostered by knowing that the new behavior has positive outcomes as opposed to the negative outcomes that accompany the current behavior; and planning (action planning and coping planning) which serves as an operative mediator between intentions and behavior. The quit rates at 3 months were 9.4% (on-site counselling) and 11.5% (SMS) compared with 9.3% in the control group (p = 0.93) (Schwarzer, 2008). The Quit to Win Contest 2013 tested the effectiveness of combining competition and short-term monetary incentives to motivate smokers to quit smoking. The overall quit rate was 9.4% (95% CI 7.8%-11.4%). The quit rate for participants who were given prior notice about receiving the monetary incentive (HKD500) upon validated abstinence at the 3-month follow-up was 9.0% (95% CI 6.4-12.6%), which was similar to those who received a delayed notice (Quit rate=10.9%, 95% CI 8.1%-14.7%) and those who did not receive any incentive for abstinence (Quit rate=8.4%, 95% CI 5.9%-11.9%). In 2014, an RCT tested the effectiveness of the cut down to quit (CDTQ) and quit immediately (QI). Smoking reduction or cut down to quit (CDTQ) approach is an important alternative strategy for promoting smoking cessation with several RCTs showing effectiveness (Batra et al., 2005; Carpenter, Hughes, Solomon, & Callas, 2004; Shiffman, Ferguson, & Strahs, 2009). In contrast, a meta-analysis of 10 trials found similar effects in quit rates when comparing quit immediately vs. cut down to quit (15% vs. 14.1%). Some further suggests that quitting gradually was not associated with less success than quit immediately (Lindson-Hawley, Aveyard, & Hughes, 2013). The overall self-reported quit-rate at 6-month is 10.9%. In the last Quit-to-Win Contest in 2015, a 3-armed RCT tested the effectiveness of the active referral and AWARD approaches. The preliminary result presented that the active referral intervention resulted in better quitting.

In QTW Contest 2016, we evaluated the effects of a higher intensity and personalized active referral (HAR) vs. low intensity text messaging (SMS) vs. very brief SC advice (VBA; control group) on encouraging smoking cessation (SC) service use and

increasing the quit rate. HAR included onsite AR, brief advice using AWARD model with a warning leaflet plus a referral card provided, and instant messages as reminders. Text messaging group included brief advice using AWARD model with the same warning leaflet plus the referral card provided and low intensity text messages as reminders. VBA included onsite general brief advices and a 12-page booklet. Findings at 6-month follow-up of this RCT showed that the intervention group with HAR had a significantly higher self-reported quit rate than VBA (the control group) (17.0% vs. 11.2%, P = 0.02). Text messaging group also had significantly higher self-reported quit rate than the control group (17.1 % vs. 11.2%, P = 0.02).

The seven Quit to Win Contests in Hong Kong recruited over 8,000 smokers from the community. The competition probably helped in boosting up participants' confidence and motivation to quit but additional brief counselling and short messaging services did not significantly increase the quit rate. Lucky draws were included in the past contest for participants who successfully quit (validated by biochemical tests). In accordance with the research direction suggested by the above foreign studies, the forthcoming RCT on Quit to Win Contest will examine the effectiveness of (1) personalized active referral to smoking cessation (SC) services and (2) text messaging on encouraging SC services use to increase quitting compared with control subjects among current smokers who joined the contest.

Community participatory model for smoking cessation

Community-Based Participatory Research (CBPR) is a partnership approach in scientific research that involves the collaboration among community partners and academic researchers throughout the research process (Israel, Schulz, Parker, & Becker, 1998). It has been found effective in enhancing community input, building community capacity, and addressing barriers to health in study participants who have historically been underrepresented in research (Andrews, Newman, Heath, Williams, & Tingen, 2012). Community partners have the capability of mobilizing local social resources and manpower and utilizing their network within the community, which is beneficial to a scientific research involving population-based interventions. To effectively raise the awareness of the contest and recruit as many participants as we can from the community, working with NGOs in the 18 Hong Kong districts with a CBPR model should be one effective way of program implementation.

The challenge of applying the CBPR model in the smoking cessation program is to equip the staff from NGOs and HKU about the related skills and knowledge and maintain the quality of research process and intervention. Process evaluation is a systematic procedure during the delivery of public health interventions to understand how well the program does and to link the progress to outcomes (Centers for Disease Control and Prevention, 2008). In addition to the training programme and briefing session to be provided to the participating NGOs, monitoring and documentation are needed throughout the recruitment and research process so that the quality and integrity of the effort by the involved NGOs can be evaluated.

Rationale of using active referral approach

Smoking cessation substantially increases quit rate and WHO has urged to promote smoking cessation services (World Health Organization, 2015). Smoking cessation services in Hong Kong are under-used with most of the adult daily smokers (79.6%) who had never used smoking cessation services (Census and Statistics Department, 2016).Among these smokers, only 2.4% were willing to use the services. Our previous RCT in previous QTW Contest 2015 evaluated the effects of low-intensity active referral (LAR) vs. very brief general SC advice (VBA) on quitting. LAR included onsite AWARD counselling and collection of smokers' personal contact information for SC services providers to connect with the smokers. Findings at 3-month follow-up of this RCT suggested the LAR intervention resulted in significantly higher self-reported quit rate than VBA in the control group (18.7% vs. 14.0%. P<0.001). Our RCT in previous QTW Contest 2016 evaluated the effects of a higher intensity and personalized active referral (HAR) vs. low intensity text messaging (SMS) vs. very brief SC advice (VBA; control group) on encouraging smoking cessation (SC) service use and increasing the quit rate. Findings at 6-month follow-up of this RCT showed that the intervention group with HAR had a significantly higher self-reported quit rate than VBA (the control group) (17.0% vs. 11.2%, P = 0.02). Text messaging group also had significantly higher self-reported quit rate than the control group (17.1 % vs. 11.2%, P = 0.02).

However, these SMS-based RCTs could not provide real-time responses from the counsellors, which might weaken the smokers' intention to quit and lower the intensity of social support. In the iQuit RCT, around 18% of smokers from the

intervention group discontinued the programme (Naughton et al., 2014). In the present proposal, we hope to enrich the SMS-based intervention by using WhatsApp or WeChat, which can provide an interactive platform and develop a semi-personalized interactive IM system that can tailor for the smokers according to their characteristics, needs and demand.

Therefore, the present study will examine (1) the effectiveness of regular messages and semi-personalized instant messaging with AWARD brief advice and active referral to smoking cessation (SC) services and (2) explore the use of CBPR model to build capacity and to engage community partners in taking on this important public health issue for sustainability in the community. In addition, a process evaluation will be conducted to assess the effectiveness of the recruitment activity and how it is linked with the overall program outcomes.

8. Study design

The present study consists of three phases: (I) provision of the training for smoking cessation ambassadors; (II) process evaluation of participant recruitment activities; and (III) a cluster RCT to test the effectiveness of a combined intervention using brief cessation advice, active referral and semi-personalised interactive instant messaging compared with control subjects among current smokers who joined the contest.

8.1 Selection of subjects

Phase I

Around 100 staff/helpers from the district partners, COSH, HKU and other parties who will take part in the QTW 2017 will be invited to participate in a brief training programme. The TTT programme will (1) provide staff/ helpers with an overview of the QTW 2017 project; (2) equip them with the knowledge and new simple skills of helping smokers stop smoking in their respective communities; and (3) train them to give brief advice on smoking reduction and use of existing smoking cessation services. The effect of the programme will be evaluated by pre- and post-tests. At least two half-day sessions of training will be held and the following topics will be covered in each session:

(1) Introduction of COSH;
(2) Objective and details of the QTW 2017 Campaign;
(3) Marketing and promotion of smoke-free messages;

(4) Global tobacco epidemic and control measures, including smoke-free policies and smoking cessation services in Hong Kong;
(5) Smoking hazards of active smoking, second-hand and third-hand smoke;
(6) Effectiveness of instant messaging interaction, brief smoking cessation interventions, referral to and use of existing services;
(7) Brief smoking cessation and reduction advice and counselling skills with case study and sharing; and
(8) A brief introduction of evaluation using randomised controlled trial (RCT).

Phase II

About 70 sessions of recruitment and promotion activities will be organised with liaison with COSH aiming to recruit 1,200 participants. Well-trained smoking cessation ambassadors (about 6 per recruitment activity) will be deployed for onsite recruitment, brief intervention (e.g. brief SC advice), monitoring and evaluation. Each recruitment activity will be a study unit of the process evaluation and all the recruitment inputs and outcomes will be documented by a research staff for further analysis.

Phase III

We use the best study design possible under the constraints of the QTW to evaluate the effectiveness of two new brief interventions. We follow the CONSORT (Schulz, 2010) in the design, implementation and reporting for the proposed cluster RCT. Participants will be recruited in the community of all 18 districts during QTW recruitment activities in Hong Kong.

The cluster RCT has 2 arms: intervention group A (Group A), and control group (Group B). The participants will be randomly assigned to one of the 2 groups based on the session they are being recruited (cluster randomization). Individual randomization at the recruitment site can have several subjects being recruited together at the same time resulting in contamination as some subjects may be exposed to the unintended interventions inadvertently. Based on the method of random assignment of recruitment session, subjects will be allocated according to the randomly selected session to either group. The randomization for group assignment will be generated by the investigators of the project before participant's recruitment

and allocation concealment will be ensured (please refer to "Randomization" for details). We follow the CONSORT (Schulz, Altman, Moher, & Consort Group, 2011) in the design, implementation and reporting for the proposed cluster RCT.

Individual telephone or face-to-face interviews will be conducted on a purposive sample of quitters and non-quitters participated in the RCT after completion of 6-month follow-up to explore their perceptions on the intervention, comments of improvement and factors contributing to the quitting/ non-quitting. The study endpoint (sample size) will be determined by data saturation.

<u>Inclusion criteria</u>

- Hong Kong residents aged 18 or above
- Smoke at least 1 cigarette per day in the past 3 months
- Able to communicate in Cantonese (including reading Chinese)
- Exhaled carbon monoxide (CO) 4 ppm or above, assessed by a validated CO Smokerlyzer.
- Intent to quit / reduce smoking
- Using a cell phone with instant messaging tool (e.g. WhatsApp, WeChat)
- Able to use instant messaging tool (e.g. WhatsApp, WeChat) for communication

<u>Exclusion criteria</u>

- Smokers who have communication barrier (either physically or cognitively)
- Have participation in other smoking cessation programmes or services

8.2 Procedures

Phase I: Develop a smoking cessation training curriculum for the staff from NGOs and HKU

The smoking cessation training curriculum will be designed to illustrate the psychological and behavioral therapies in managing the care of the smokers. Throughout the training, participants will be taught a variety of topics by trained nurses and trained smoking cessation counsellors including: (1) Introduction of the Quit-to-Win Contest & the RCT; (2) Smoking trend and knowledge of health hazards from smoking; (3) COSH and social marketing on smoking cessation; (4) Recruitment strategies: How to approach smokers; (5) Experience sharing in communication with

smokers; (6) Assessment of quitting readiness and individualized brief counselling; and (7) Technical skills such as conducting surveys and use of Smokerlyzers and advice on smoking reduction. At the end of the program, participants should be capable of delivering brief advice of smoking cessation for smokers. Upon completion of the training program, a certificate of attendance will be awarded to each participant. The outcomes of the training will be evaluated through a self-administered survey before, immediate after, and 6 months post-training, which includes knowledge, attitudes, and practice of smoking cessation intervention.

Phase II: Process evaluation of the recruitment activities

Quality assurance

Throughout the process evaluation, the trained staff from HKU and NGOs will be monitored on site. Spot checks will be conducted at every venue by an investigator or a more senior research assistant to ensure a consistent delivery of the interventions proposed. They will also be responsible to monitor the whole process of each recruitment activities, and record necessary information related to the recruitment input, outcome and other environmental factors. Recruitment input includes the number of recruitment workers, posters and leaflets used. Recruitment outcome includes the number of smokers and non-smokers approached and the number of people who pay attention to the recruitment booth. The research staff will also assess the environmental factors including weather, date, time, location, facilities that may have an impact on the achievement of the recruitment sessions.

Phase III: Recruit and provide smoking reduction/cessation counselling

At the recruitment sessions, smoking cessation counsellor will measure the potential participant's level of carbon monoxide (CO) in exhaled air, screen their eligibility for entering the contest, and provide the self-help smoking cessation booklets developed by the Hong Kong Council on Smoking and Health (COSH). Then the counsellor will explain and invite the participants to join the RCT on smoking cessation intervention. Written consent for voluntary participation in the trial will be obtained before administering the baseline questionnaire and delivery of the intervention for the participants.

Hypothesis

The major research hypothesis is: "Cocktail" intervention of brief advice, instant messaging and active referral (AIR) group (Group A) will have higher smoking cessation rate than the control group (Group B). The other hypotheses for Group A vs.

vs. Group B that the former will have higher services use rate, validated smoking cessation rate and smoking reduction rate.

Quit to Win Contest

In the Quit to Win study, two-arm RCT will be conducted to test the effectiveness of "cocktail" intervention of brief advice, instant messaging and active referral (AIR) SC services use in achieving smoking abstinence. A detailed flow chart of the RCT is attached (Appendix 1).

Intervention group

Smokers in intervention groups will receive a combined "cocktail" intervention of (i) brief advice, (ii) instant messaging and (iii) active referral.

(i) Brief intervention using AWARD model (Group A)

Smokers will receive AWARD intervention delivered by the SC counsellors who completed the TTT programme and a brief warning leaflet emphasising on smoking harms (especially one out of two smokers will be killed by smoking, and the risk could be up to 2/3 for high risk smokers) and benefits of cessation.

AWARD model

AWARD will be delivered to smokers onsite and this includes: **A**sk about smoking history, **W**arn about the high risk *(with a brief health warning leaflet, see below for information)*, **A**dvise to quit as soon as possible and not later than a quit date (which will qualify them for the QTW prizes), **R**efer smokers to smoking cessation services *(with a referral card, see below for information)*, and **D**o it again: to repeat the intervention; participants who fail to quit or relapse will be encouraged to quit again (and those who have quit will be encouraged to prevent relapse) during each telephone follow-up. The whole process of AWARD can be delivered within 30 seconds to 1 minute.

a) Brief leaflet on health warning and smoking cessation

The 2-side colour printed A4 leaflet, which systematically covers the most important messages to motivate smoking cessation and being used in QTW 2015, will be disseminated to smokers during the counselling, with improvements if necessary.

b) Referral card

The 3-folded "Smoking Cessation Services" card which was developed and used since QTW Contest 2015 will be disseminated to smokers in this project, with improvements if necessary. The content consists of brief information and highlights of existing smoking cessation services, contact methods, motivation information and strong supporting messages or slogans.

(ii) Semi-personalized instant messaging interaction

Similar to QTW2016, regular messages will be sent via instant messaging (IM) services (e.g. WhatsApp, WeChat) since initial contact and until 3-month after baseline with a tapering schedule:

- Baseline to 4-week (1-month): 2 times/ week (8 in total)
- 4-week to 8-week (2-month): 3 times/ 2 weeks (6 in total)
- 8-week to 13-week (3-month): 1 time/week (5 in total)

SC messages will generally include benefits of SC, encouragement on abstinence and use of SC services, tips on avoiding/ handling craving and reminder of participating in telephone follow-up. An IM message will also be sent at 26-week as a reminder of 6-month follow-up, making up a total of 20 regular messages.

In addition to sending regular messages, semi-personalized instant messaging (IM) interactions (e.g. WhatsApp, WeChat) will be developed and smokers can communicate with counsellors in real-time during office hours (0930 to 1830 every working day) since initial contact and until 3-month after baseline. Our counsellors will trigger the conversation and invite the smokers to response. Semi-personalized conversation will be developed according to smokers' basic characteristics and intention to quit and use existing SC services. Through real-time online chatting, smokers can acquire SC information immediately and gain social support. Counsellors can also keep contact with the smokers to offer help, positive encouragement, reflections and reminders about SC according to smokers' personal needs and to encourage them to seek SC services. Details of successfully booked SC appointment (e.g. SC services address, contact information, date, appointment number etc.) will be delivered to the smokers using IM. All smokers will receive a reminder-to-attend IM messages 1-3 days before the appointment date. The details of IM

development is described below. We have extensive experience in IM and SMS designed for smokers.

(iii) Active referral to SC services

Similar to QTW Contest 2016, smokers in this project will be introduced to various SC services in Hong Kong (using the referral card) and be motivated to use the services. Interventions in this group are designed to promote using the existing SC services to increase abstinence rate. Cochrane review suggests that more intensive contact with smokers is the way to increase participation in smoking cessation programmes (Belisario, Bruggeling, Gunn, Brusamento, & Car, 2012).

Research staff will monitor the use of SC services by smokers at each follow-up (1, 2, 3 & 6-month) and assist participants to book or re-book appointments if necessary. We shall liaise with the existing service providers and seek their assistance in helping the smoker in a timely manner.

Control Group: very brief SC advice (Group B)

Participants in the control group will receive minimal intervention, including: (1) Placebo short-message service (SMS) reminder messages to participate in telephone follow-up (4 in total; 1 each for 1-, 2-, 3- and 6-month follow-up) ; (2) very brief, minimal and general smoking cessation advice, such as “Please quit smoking for improving health and save money”, “Please refer to the booklet for the details about smoking cessation” and “Please call us if you have any enquiry”.

Subjects in the both intervention and control group will also receive a 12-page generic self-help SC booklet provided by COSH (**https://tinyurl.com/ydy3xssu**).

Table 1. Summary of intervention in 2 groups

	Intervention (Group A)	Control (Group B)
Instant messaging support + regular messages	✓	
Active referral to SC service	✓	
AWARD advice + warning leaflet + referral card	✓	

Placebo SMS messages		✓
Very brief SC advice		✓
12-pg COSH booklet	✓	✓

Non-trial Group

Those who are unable to read or communicate using Chinese, or those who refuse to participate in the RCT, can still participate in the QTW Contest and will receive the same monetary incentive if he/she passes the biochemical validations at 3- and 6-month. This group will be analysed separately from the RCT.

Follow-up

All participants in the RCT will be followed-up at 1-, 2-, 3- and 6-months by telephone interview to assess their smoking status and quitting progress.

For those unreachable participants at the schedule follow-up time, we shall make further calls, but limited to a maximum of 7 calls and 1 voice message as a reminder for their quitting. Self-reported quitters (no smoking in past 7 days) at 3- and 6- month follow-up will be invited for biochemical and non-biochemical validation. Biochemical validation includes the measurement of exhaled CO level and saliva cotinine level of the participants, which will be conducted by research assistants. The non-biochemical validation includes asking the quitter a few questions on the consequences of quitting, confirming quitter's quitting status by family members and assessment on the quitter by the interviewer. These are broadly similar to previous QTW Contests.

8.3 Randomization

By cluster randomization, all participants recruited in a particular recruitment session (one day may have more than one activity) will be allocated to one of the RCT groups. Block randomization will be used to allocate the recruitment activity into the two RCT groups to ensure the number of recruitment activities for the two RCT groups is similar. The numbers for the permutation in the blocks will be generated with the website http://www.random.org (a website for generating random integers), and then merge with the list of all recruitment days.

8.4 Instruments

Phase I

A course evaluation form and a self-administered questionnaire including knowledge, attitude, and practice of smoking cessation will be completed by the participants of the training workshops (Appendix 2).

Phase II

A process evaluation form will be used to record the recruitment outcomes and observations including the number of people reached in the recruitment sessions of all recruitment sessions. It will be administered by the investigators or research staff.

Phase III

Quit to Win

Three sets of questionnaires will be adapted from our previous Quit to Win Contest conducted in 2015. These include: (1) a baseline questionnaire which collects demographic data, smoking behavior, quit attempts, smoking-related psychological factors and perceived social support when they participated the Contest (Appendix 8); (2) a set of follow-up questionnaires 1 month & 2 month booster interventions (Appendix 9a-b and 10a-b); and (3) a set of follow-up questionnaires for 3- & 6-months (Appendix 11a-b and 12a-b) which collects information on smoking behavior, quit attempts, smoking-related psychological factors and perceived social support in the quitting process, as well as the impact of the Quit to Win Contest. An interview guide will be developed for qualitative interviews with quitters and non-quitters.

8.5 Sample size

Phase I

All staff/helpers from the participated NGOs and HKU who participate in the recruitment will be invited to attend the training program. A total of 100 participants (including a minimum of 36 NGO staff and HKU student helpers) can join the smoking cessation training program.

Phase II

COSH targeted to organize at least 2 recruitment sessions in each of the 18 districts in Hong Kong. There will be about 70 recruitment sessions to be evaluated.

Phase III

Quit to Win

The sample size calculation is based on the primary outcome of biochemically validated 7-day point prevalence quit rate at the 6-month follow-up. Result from QTW2015 showed that the intention-to-treat biochemically validated abstinence at 6-month was 5.1%. Based on a meta-analysis of mobile health intervention for smoking

cessation, the intervention effect on validated quit rate at 6-month has an RR of 1.83 (Whittaker, McRobbie, Bullen, Rodgers, & Gu, 2016). To detect a significant difference of quit rate between Group A and Group B with a power of 80% and 5% significant level, we will need 586 subjects per group. The design effect is estimated to be negligible, based on the results from QTW2010 which shows an intra-cluster correlation of <0.001 for biochemically validated abstinence at 6-month (Chan et al. 2017). Total sample size needed = 586 × 2 = 1,172.

For the post-intervention qualitative study, a purposive sample of quitter and non-quitter will be interviewed with sample size determined by data saturation.

8.6 Statistical Analysis

Data will be entered into SPSS for Windows (version 23). A logic check program will be installed for entry validation. Descriptive statistics such as frequency, percentage, and mean will be used to summarize the outcomes and other variables. Chi-square tests and t-tests will be used to compare outcome variables between subgroups. Generalized Estimating Equation (GEE) models will be applied to test the intervention effect, to identify the baseline predictors of successful quitting and to assess the changes in smoking-related factors over time. The intention-to-treat (ITT) analysis will be used such that those lost to contact and refused cases at the follow-ups will be treated as a failure to achieve any cessation outcome. Complete case analysis will also be conducted after excluding participants with missing data.

8.7 Chronological outline of research plan

Timeline	2017									2018				
Preparation														
**1. Preparation of IRB, training material, smoking cessation materials and research instruments (1.5m)**														
Intervention														
Phase I & II														
**2. Training of smoking cessation counsellors and logistics arrangement (1m)**														
**3. Evaluation survey (Pre-training, immediate post-training) (2m)**														

**4. Data analysis and report preparation for the smoke-free Training (2m)**														
Phase III														
**5. Individual interview (1m)**														
**Phase IV**														
**6. Recruitment of subjects at the districts & NGOs (3.5m)**														
**7. 1 month telephone follow-up (booster for Group A & B) (3.5m)**														
**8. 2 month telephone follow-up (for intervention group) (3.5m)**														
**9. 3 months telephone follow-up and biochemical validation of quitters (4m)**														
**10. 6 months telephone follow-up and biochemical validation of quitters (4m)**														
**Analysis and write up**														
**11. Data collection and cleaning (10m)**														
**12. Data analysis (7m)**														
**13. Report preparation and result dissemination (6m)**														

9. Drug investigation: Nil

10. Describe any unusual or discomforting procedures to be used: Nil.

11. Are there any hazards associated with the investigation? No

12. Direct access to source data/documents

The raw data will be stored on an external hard-disk and locked in a cupboard with keys kept by the Principal Investigator. Only the Investigators and Research Assistant of the project will be permitted to access the raw data and/or study records. The data will be scanned and kept for 10 years or longer after the study is completed. Individual participants will not be directly identifiable from the dataset to be used for analysis.

13. Dissemination of study result

The research findings will be reported to the COSH for policy evaluation, disseminated in local and international conferences, and published in international peer-reviewed journals.

14. Consent

Participation in the study is totally voluntary. The smoking counselors at the study sites will explain to smokers who agree to join the Quit to Win Contest by COSH that we are carrying out a study on smoking cessation with more incentives than the lucky draw for the grand prizes, but the smokers will not be informed about the specifics of the incentives. The smoking counsellors will explain to the participants that they will receive telephone calls at 1-, 2-, 3- & 6-months for the follow-up of their smoking status. The participants will be assured that they can withdraw from the study anytime without any prejudice, and all the information will be kept confidential and results will be reported in an aggregate format. Agreement to participate in the RCT will be considered as consent and participants are required to sign the written consent form.

15. Conflict of interest: None

16. Financing and insurance

Research Fund: The Hong Kong Council on Smoking and Health

Indemnity and Compensation: Nil.

References

Andrews, J. O., Newman, S. D., Heath, J., Williams, L. B., & Tingen, M. S. (2012). Community-based participatory research and smoking cessation interventions: a review of the evidence. *Nurs Clin North Am, 47*(1), 81-96. doi:10.1016/j.cnur.2011.10.013

Batra, A., Klingler, K., Landfeldt, B., Friederich, H. M., Westin, A., & Danielsson, T. (2005). Smoking reduction treatment with 4-mg nicotine gum: a double-blind, randomized, placebo-controlled study. *Clin Pharmacol Ther, 78*(6), 689-696. doi:10.1016/j.clpt.2005.08.019

Belisario, J. S. M., Bruggeling, M. N., Gunn, L. H., Brusamento, S., & Car, J. (2012). Interventions for recruiting smokers into cessation programmes. *Cochrane Database of Systematic Reviews*(12). doi:10.1002/14651858.CD009187.pub2

Cahill, K., & Perera, R. (2008). Quit and Win contests for smoking cessation. *Cochrane Database Syst Rev*(4), CD004986. doi:10.1002/14651858.CD004986.pub3

Cahill, K., & Perera, R. (2011). Competitions and incentives for smoking cessation. *Cochrane Database Syst Rev*(4), CD004307. doi:10.1002/14651858.CD004307.pub4

Carpenter, M. J., Hughes, J. R., Solomon, L. J., & Callas, P. W. (2004). Both smoking reduction with nicotine replacement therapy and motivational advice increase future

cessation among smokers unmotivated to quit. *J Consult Clin Psychol, 72*(3), 371-381. doi:10.1037/0022-006X.72.3.371

Census & Statistics Department. (2001). *Hong Kong Annual Digest of Statistics 2001*. Hong Kong SAR.

Census and Statistics Department. (2016). *Thematic Household Survey, Report No.59: Pattern of Smoking*. Hong Kong SAR: Hong Kong SAR Government.

Centers for Disease Control and Prevention. (2008). Introduction to Process Evaluation in Tobacco Use Prevention and Control. .

Chan, S. S. C. (2011). *Smoking Cessation Thru Quit to Win Contest 2009: Experience and effectiveness*. Paper presented at the 5th Cross-Strait Conference on Tobacco Control, Taipei.

Chan, S. S. C., Cheung, Y. T. D., Wong, Y. M. B., Kwong, A., Lai, V., & Lam, T. H. (2017). A Brief Smoking Cessation Advice by Youth Counselors for the Smokers in the Hong Kong Quit to Win Contest 2010: a Cluster Randomized Controlled Trial. *Prevention Science* (online first). PMID28755244

Israel, B. A., Schulz, A. J., Parker, E. A., & Becker, A. B. (1998). Review of community-based research: assessing partnership approaches to improve public health. *Annu Rev Public Health, 19,* 173-202. doi:10.1146/annurev.publhealth.19.1.173

Lam, T. H. (2012). Absolute risk of tobacco deaths: one in two smokers will be killed by smoking: comment on "Smoking and all-cause mortality in older people". *Arch Intern Med, 172*(11), 845-846. doi:10.1001/archinternmed.2012.1927

Lam, T. H., Ho, S. Y., Hedley, A. J., Mak, K. H., & Peto, R. (2001). Mortality and smoking in Hong Kong: case-control study of all adult deaths in 1998. *BMJ, 323*(7309), 361.

Lindson-Hawley, N., Aveyard, P., & Hughes, J. R. (2013). Gradual reduction vs abrupt cessation as a smoking cessation strategy in smokers who want to quit. *JAMA, 310*(1), 91-92. doi:10.1001/jama.2013.6473

McGhee, S. M., Ho, L. M., Lapsley, H. M., Chau, J., Cheung, W. L., Ho, S. Y., . . . Hedley, A. J. (2006). Cost of tobacco-related diseases, including passive smoking, in Hong Kong. *Tob Control, 15*(2), 125-130. doi:10.1136/tc.2005.013292

Schwarzer, R. (2008). Modeling Health Behavior Change: How to Predict and Modify the Adoption and Maintenance of Health Behaviors. *Applied Psychology, 57*(1), 1-29. doi:10.1111/j.1464-0597.2007.00325.x

Shiffman, S., Ferguson, S. G., & Strahs, K. R. (2009). Quitting by gradual smoking reduction using nicotine gum: a randomized controlled trial. *Am J Prev Med, 36*(2), 96-104 e101. doi:10.1016/j.amepre.2008.09.039

Whittaker, R., McRobbie, H., Bullen, C., Rodgers, A., & Gu, Y. (2016). Mobile phone-based interventions for smoking cessation. *Cochrane Database Syst Rev. 4,* CD006611

Wong, D. C. N., & Chan, S. S. C. (2012). *Effectiveness of a community-based brief smoking cessation intervention through the Quit to Win Contest: a randomized controlled trial.* Paper presented at the 15th World Conference on Tobacco or Health, Singapore.

World Health Organization. (2015). Policy recommendations for smoking cessation and treatment of tobacco dependence. Retrieved from http://www.who.int/tobacco/publications/smoking_cessation/recommendations/en/
