# Supplementary material for: Engagement With a Mobile Chat-Based Intervention for Smoking Cessation: A Secondary Analysis of a Randomized Clinical Trial
Source: JAMA Netw Open. 2024 Jun 26;7(6):e2417796. doi: 10.1001/jamanetworkopen.2024.17796 (PMC11208971; doi:10.1001/jamanetworkopen.2024.17796)
Supplement: Supplement 2. — eFigure. Participant Flow Diagram eTable 1. Baseline Characteristics of All Randomized Participants eTable 2. Model Selection Results eTable 3. Model Diagnostics eTable 4. Retention Rate by Engagement Trajectories eTable 5. Associations of Engagement Trajectories With Smoking Abstinence eTable 6. Associations of Engagement Trajectories With Smoking Abstinence by Complete Case Analyses [file jamanetwopen-e2417796-s002.pdf]

Supplementary Online Content

Li Y, Luk TT, Cheung DYT, et al. Engagement with a mobile chat-based intervention for smoking cessation: a secondary analysis of a randomized clinical trial. *JAMA Netw Open*. 2024;7(6):e2417796. doi:10.1001/jamanetworkopen.2024.17796

eFigure. Participant Flow Diagram

eTable 1. Baseline Characteristics of All Randomized Participants

eTable 2. Model Selection Results

eTable 3. Model Diagnostics

eTable 4. Retention Rate by Engagement Trajectories

eTable 5. Associations of Engagement Trajectories With Smoking Abstinence

eTable 6. Associations of Engagement Trajectories With Smoking Abstinence by Complete Case Analyses

This supplementary material has been provided by the authors to give readers additional information about their work.

© 2024 Li Y et al. *JAMA Network Open.*

eFigure. Participant flow diagram

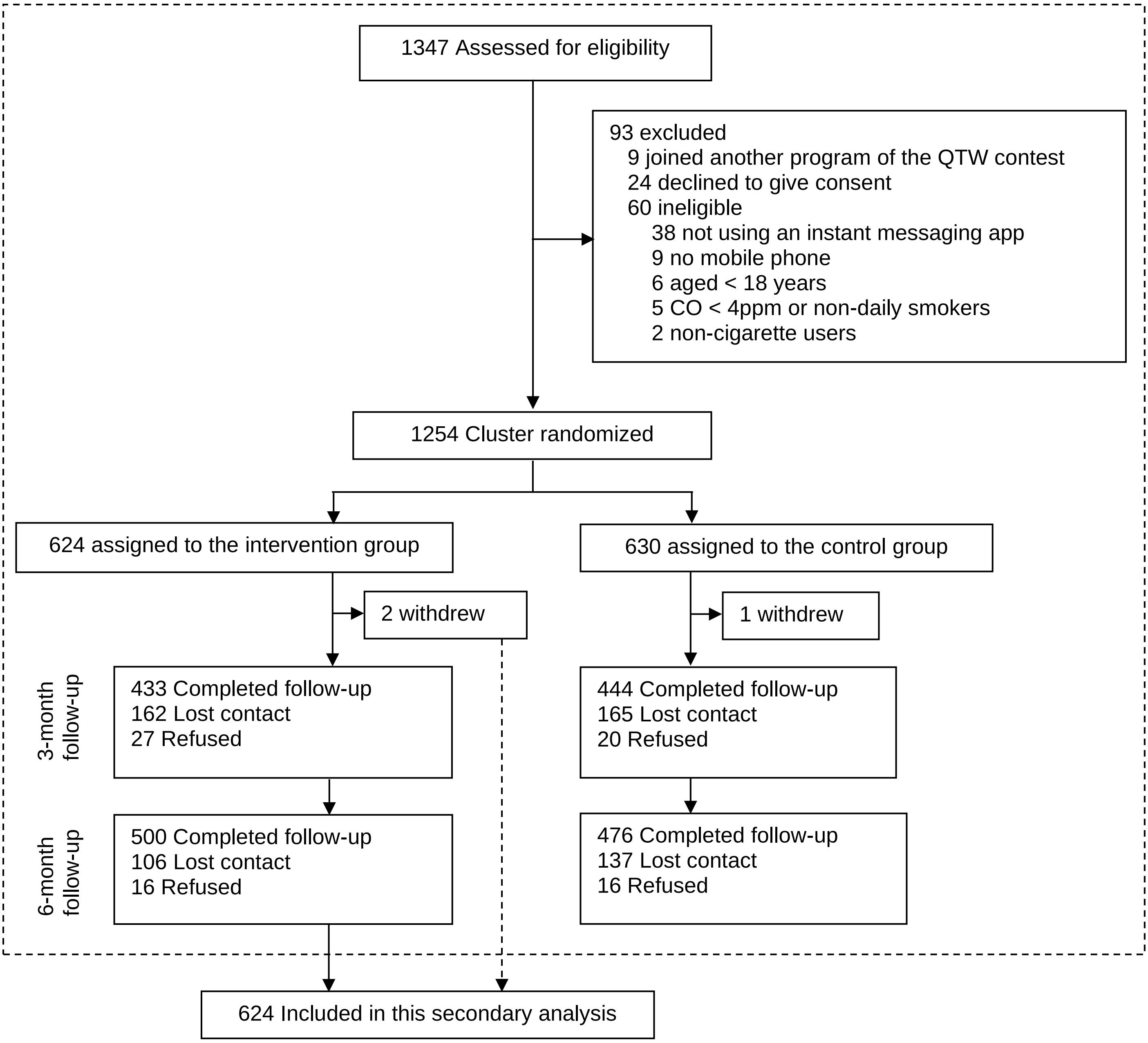

© 2024 Li Y et al. *JAMA Network Open.*

eTable 1. Baseline characteristics of the Quit to Win study

Characteristics	No. (%)		
	Total (N=1254)	Intervention (n=624)	Control (n=630)
Sex			
Male	977 (77.9)	479 (76.8)	498 (79.1)
Female	277 (22.1)	145 (23.2)	132 (21.0)
Age, mean (SD), y	42.8 (16.5)	42.1 (16.2)	43.4 (16.7)
Age group, years			
18-30	239 (19.1)	125 (22.6)	114 (21.2)
30-39	268 (21.3)	140 (25.3)	128 (23.9)
40-49	238 (19.0)	125 (22.6)	113 (21.0)
≥ 50	346 ((27.6)	164 (29.6)	182 (33.9)
Educational level			
Primary or below	68 (5.4)	26 (6.6)	42 (10.6)
Secondary	548 (43.7)	284 (72.1)	264 (66.8)
Tertiary	173 (13.8)	84 (21.3)	89 (22.5)
Daily cigarette consumptions, Mean (SD)	13.9 (8.7)	13.8 (8.3)	13.9 (9.0)
Nicotine dependence[a]			
Low	602 (51.3)	295 (50.8)	307 (51.9)
Moderate / High	571 (48.7)	286 (49.2)	285 (48.1)
Intention to quit in 30 days			
No	772 (64.3)	377 (62.8)	395 (65.7)
Yes	429 (35.7)	223 (37.2)	206 (34.3)
Past quit attempt			
Never	583 (47.3)	273 (43.8)	320 (50.8)
Beyond past 12 months	449 (35.8)	237 (38.0)	212 (33.7)
Within past 12 months	212 (16.9)	114 (18.3)	98 (15.6)
Perceptions of quitting, mean (SD)[b]			
Importance	7.1 (2.1)	7.2 (2.1)	7.0 (2.1)
Difficulty	7.0 (2.3)	7.1 (2.2)	6.8 (2.3)
Confidence	5.9 (2.2)	6.0 (2.2)	5.7 (2.1)

[a] Measured by Heaviness of Smoking Index, scores ranged from 0-6, with higher scores indicating greater nicotine dependence
[b] Scores ranged from 0-10, with higher scores indicating higher perceived importance, difficulty, or confidence.

© 2024 Li Y et al. *JAMA Network Open.*

eTable 2. Model selection results

**Number of trajectories**	**Polynomial order[a]**	**BIC (n=7488)[b]**	**BIC (n=624)[c]**
1	3	-2970.69	-2965.72
2	2 1	-1895.97	-1888.51
3	1 2 2	-1822.25	-1809.82
4	2 1 1 1	-1806.98	-1792.07
5	1 1 1 2 1	-1809.38	-1790.74

[a] Trajectory shapes; 1=linear; 2=quadratic; 3=cubic.
[b] BIC = Bayesian information criterion (for the total number of observations).
[c] BIC = Bayesian information criterion (for the total number of participants).

© 2024 Li Y et al. *JAMA Network Open.*

eTable 3. Model diagnostics[a]

Engagement trajectories	AvePP[b]	OCC[c]	$\|\pi\text{-}P\|$[d]
Low engagement	0.95	7.35	0.03
Rapid-declining	0.89	52.15	0.03
Gradual-declining	0.86	58.26	0.001
High engagement	0.92	198.3	0.004

[a] Model polynomial order: 2111
[b] Average group posterior probability. An AvePP greater than 0.7 for all groups is recommended.
[c] Odds of correct classification. An OCC of 5 or more is recommended for all groups.
[d] π: estimated group probabilities; P: proportion assigned to the group using the maximum probability rule.

© 2024 Li Y et al. *JAMA Network Open.*

eTable 4. Retention rates by engagement trajectories

Engagement trajectories	6-mo follow-up		3-mo follow-up	
	No./ total No. (%)	*P* value[a]	No. / total No. (%)	*P* value[a]
Low engagement	334/447 (74.7)	<.001	269/447 (60.2)	<.001
Rapid-declining	81/86 (94.2)		79/86 (91.9)	
Gradual-declining	54/58 (93.1)		55/58 (94.8)	
High engagement	31/33 (93.9)		30/33 (90.9)	

[a] *P* values were calculated by Chi-Square test

© 2024 Li Y et al. *JAMA Network Open.*

eTable 5. Associations of engagement trajectories with smoking abstinence[a]

Variable	6-mo follow-up			3-mo follow-up		
	No./ total No. (%)	Model 1[b] Adjusted RR (95% CI)	Model 2[c] Adjusted RR (95% CI)	No./ total No. (%)	Model 1[b] Adjusted RR (95% CI)	Model 2[c] Adjusted RR (95% CI)
Biochemically validated abstinence						
Low engagement	16/447 (3.6)	1 [Reference]	1 [Reference]	13/447 (2.9)	1 [Reference]	1 [Reference]
Rapid-declining	13/86 (15.1)	3.13 (1.31 – 7.46)	2.85 (1.18 – 6.93)	13/86 (15.1)	3.67 (1.39 – 9.71)	3.19 (1.17 – 8.64)
Gradual-declining	16/58 (27.6)	4.23 (1.76 – 10.18)	3.63 (1.45 – 9.11)	13/58 (22.4)	4.28 (1.58 – 11.61)	3.58 (1.27 – 10.10)
High engagement	8/33 (24.2)	3.48 (1.23 – 9.80)	2.99 (1.02 – 8.76)	11/33 (33.3)	6.40 (2.21 – 18.52)	5.24 (1.73 – 15.88)
Self-reported 7-day point prevalence abstinence						
Low engagement	36/447 (8.1)	1 [Reference]	1 [Reference]	45/447 (10.1)	1 [Reference]	1 [Reference]
Rapid-declining	21/86 (24.4)	1.55 (0.85 – 2.84)	1.40 (0.75 – 2.61)	23/86 (26.7)	2.33 (1.19 – 4.59)	1.92 (0.95 – 3.89)
Gradual-declining	16/58 (27.6)	1.90 (1.02 – 3.53)	1.63 (0.85 – 3.12)	18/58 (31.0)	2.19 (1.07 – 4.50)	1.70 (0.79 – 3.65)
High engagement	12/33 (36.4)	1.67 (0.80 – 3.50)	1.41 (0.65 – 3.06)	12/33 (36.4)	1.97 (0.86 – 4.50)	1.45 (0.60 – 3.49)

Abbreviation: RR, relative risk; CI, confidence interval

[a] Participants with missing data were imputed as smoking

[b] Adjusted for sex, age, education, nicotine dependence, intention to quit in 30 days, past quit attempt and perceptions of quitting at baseline were adjusted.

[c] Adjusted for sex, age, education, nicotine dependence, intention to quit in 30 days, past quit attempt, perceptions of quitting at baseline and use of any smoking cessation service were adjusted.

© 2024 Li Y et al. *JAMA Network Open.*

eTable 6. Associations of engagement trajectories with smoking abstinence by complete case analyses[a]

Variable	6-mo follow-up (n=500)			3-mo follow-up (n=433)		
	No./ total No. (%)	RR (95% CI)	Adjusted RR (95% CI)[b]	No./ total No. (%)	RR (95% CI)	Adjusted RR (95% CI)[b]
Biochemically validated abstinence						
Low engagement	16/334 (4.8)	1 [Reference]	1 [Reference]	13/269 (4.8)	1 [Reference]	1 [Reference]
Rapid-declining	13/81 (16.1)	3.35 (1.61 – 6.96)	2.99 (1.27 – 7.06)	13/79 (16.5)	3.41 (1.58 – 7.35)	2.99 (1.15 – 7.85)
Gradual-declining	16/54 (29.6)	6.19 (3.09 – 12.37)	4.88 (2.10 – 11.38)	13/55 (23.6)	4.89 (2.27 – 10.55)	4.09 (1.55 – 10.80)
High engagement	8/31 (25.8)	5.39 (2.31 – 12.59)	4.21 (1.54 – 11.47)	11/30 (36.7)	7.59 (3.40 – 16.94)	6.86 (2.44 – 19.26)
Self-reported 7-day point prevalence abstinence						
Low engagement	36/334 (10.8)	1 [Reference]	1 [Reference]	45/269 (16.7)	1 [Reference]	1 [Reference]
Rapid-declining	21/81 (25.9)	1.46 (0.91 – 2.35)	1.46 (0.80 – 2.66)	23/79 (29.1)	1.74 (1.05 – 2.88)	1.77 (0.90 – 3.44)
Gradual-declining	16/54 (29.6)	2.09 (1.29 – 3.39)	2.15 (1.17 – 3.94)	18/55 (32.7)	1.96 (1.13 – 3.38)	1.98 (0.98 – 4.01)
High engagement	12/31 (38.7)	2.15 (1.19 – 3.91)	1.79 (0.87 – 3.69)	12/30 (40.0)	2.39 (1.26 – 4.52)	1.98 (0.88 – 4.42)

Abbreviation: RR, relative risk; CI, confidence interval

[a] Participants with missing abstinence data were excluded

[b] Adjusted for sex, age, education, nicotine dependence, intention to quit in 30 days, and past quit attempt at baseline were adjusted.

© 2024 Li Y et al. *JAMA Network Open.*
