# Supplementary material for: Engagement With a Mobile Chat-Based Intervention for Smoking Cessation: A Secondary Analysis of a Randomized Clinical Trial
Source: JAMA Netw Open. 2024 Jun 26;7(6):e2417796. doi: 10.1001/jamanetworkopen.2024.17796 (PMC11208971; doi:10.1001/jamanetworkopen.2024.17796)
Supplement: Supplement 3. — Data Sharing Statement [file jamanetwopen-e2417796-s003.pdf]

Data Sharing Statement

Li. Engagement With a Mobile Chat-Based Intervention for Smoking Cessation. *JAMA Netw Open*. Published June 26, 2024. doi:10.1001/jamanetworkopen.2024.17796

Data
Data available: No
